# Supplementary material for: Meat Analogues: Relating Structure to Texture and Sensory Perception
Source: Foods. 2022 Jul 26;11(15):2227. doi: 10.3390/foods11152227 (PMC9367794; doi:10.3390/foods11152227)
Supplement: Supplementary file 1 [file foods-11-02227-s001.zip › foods-1801330-supplementary.pdf]

Supplementary information

Table S1. Ingredients reported on the package of the meat replacers studied in the present research.

Product	Ingredients
**Chicken pieces samples**	
Real Chicken Pieces (Albert Heijn)	100% chicken.
Albert Heijn—Pieces Like Chicken	Rehydrated vegetable protein (25% wheat, 12% pea), rehydrated wheat starch, water, sunflower oil, free-range egg white, soy protein isolate, natural flavourings, yeast extract, salt, vinegar powder, spices, preservative (lactic acid (E270)), iron, vitamin B12.
Food for Progress—Oumph the Chunk	Water, soy protein (23%), salt.
Fry's—Chicken Style Strips	Vegetable Protein (20%) (wheat, soya), vegetable oil (sunflower seed), flavouring (3%) (maize starch, yeast extract, savoury flavour), potato starch, wheat flour, thickener (methyl cellulose), salt, garlic.
Gold & Green—Pulled Oats	Water, oats (21%) (oat bran, whole grain oat flour, oat protein), pea protein (21%), fava bean protein (12%), canola oil, iodized salt (0.5%).
Greenway—Chick Pieces	Water, soy protein concentrate, rapeseed oil, herbs and spices (sugar, salt, garlic, onion, yeast extract, herbal spice extract (celery)), flavouring (carriers: maltodextrin, salt), emulsifier: lecithin (E322).
Naturli—Chick Free	Rehydrated pea protein, vegetable stock (salt, dextrose (corn), yeast extract, dried vegetables, natural flavouring), fermented dextrose.
Quorn—Chicken Pieces	Mycoprotein (94%), rehydrated free-range egg white, flavouring, firming agents: calcium chloride, calcium acetate.
Valess—Fillet Pieces	76% skim milk, texturized wheat protein, sunflower oil, thickener (calcium alginate, methyl cellulose), free-range chicken egg protein, food acid (potassium lactate), stabiliser (polyphosphate), oat fibre, flavouring, salt, iron.
Vantastic Foods—Chicken-Style Pieces	Soy protein (35%), water, wheat protein (20%), wheat starch, sunflower oil, salt, sugar, spices, chili, flavouring, gelling agent: calcium sulphate, calcium carbonate, glucose.
Vegetarische Slager—Kipstuckjes	93% soy structure (water, soy protein concentrate, salt), sunflower oil, natural aroma.
Veggie Chef—Kipstukjes	Rehydrated soy protein (93.1%), onion extract, natural flavourings (wheat), pea fibre, salt.
VegiDeli VBites—Meat-Free Chicken Pieces	Water, wheat gluten, soy protein, wheat fibre, rapeseed oil, nonhydrogenated vegetable fat (sustainable palm), yeast extract, natural flavourings, dried yeast, salt, onion powder, preservative: potassium sorbate, thickener: methyl cellulose, sugar.
Vivera—Plant Chicken Pieces	Rehydrated soy protein (93%), onion extract (3%), natural flavourings (wheat), pea fibres, salt.
Burger samples	
Ultimate Beef Burger (real beef)	99% beef, sea salt, natural aroma.
Albert Heijn—Burger Deluxe	Rehydrated vegetable protein (53% soy, 13% wheat), onion, water, vegetable oil (rapeseed, sunflower (in varying proportions)), free-range egg white powder, dried onion, spices, vinegar, yeast extract, salt, stabiliser (guar gum (E412), methyl cellulose (E461)), spices, pea starch, pea fibre, potato maltodextrin, barley malt extract, flavouring, vitamin B12, iron.

Beyond Meat—Beyond Burger	Water, pea protein isolate, expeller-pressed canola oil, refined coconut oil, rice protein, natural flavours, cocoa butter, mung bean protein, methylcellulose, potato starch, apple extract, pomegranate extract, salt, potassium chloride, vinegar, lemon juice concentrate, sunflower lecithin, beet juice extract (for colour), carrot.
Fry's—Traditional Burgers	Vegetable protein (16%) (contains soya, wheat), vegetable oil (sunflower seed), potato starch, maize starch, wheat fibre, flavourings, wheat flour (gluten), spices (contains sulphites), sea salt, thickener (cellulose extract), onion, mustard powder, natural colour (caramel IV).
Gardein—Ultimate Beefless Burger	Water, soy protein concentrate, canola oil, vital wheat gluten, onions, soy protein isolate, methylcellulose, yeast extract, malt extract (malted barley, water), onion powder, garlic powder, organic ancient grain flour (Khorasan Wheat, amaranth, Millet, quinoa), dried garlic, organic distilled vinegar, natural flavours, cane sugar, potato starch, sea salt, salt, celery seed, organic cane sugar, smoke flavour, spice extracts, spices, pea protein.
Garden Gourmet—Incredible Burger	Water, soy protein concentrate (19%), vegetable oils (rapeseed, coconut), wheat gluten, vinegar, natural flavours, garlic powder, vegetable concentrates (beetroot, carrot, pepper), salt, barley malt extract, black pepper, spirulina concentrate, stabiliser (methylcellulose).
Greenway—Burger	Water, 22% pea flour, 5% fried onion (onion, rapeseed oil), coconut fat, vegetable oils (rapeseed, sunflower), dietary fibre (bamboo, chicory), potato proteins, flavourings (natural flavour, aroma, flavouring preparation), thickeners: methyl cellulose–sodium alginate–calcium sulphate–diphosphates, maltodextrin, colouring: beetroot red, salt, yeast extract, vegetable powders (onion, carrot, tomato), herbs, spices, zinc citrate, vitamin B12, iron citrate.
Linda McCartney—Quarter Pounder	Rehydrated textured soy protein (58%), rapeseed oil, water, onion (9%), seasoning (yeast extract, malted barley Extract, onion powder, flavouring, salt, garlic powder), stabiliser: methyl cellulose, chickpea flour, flavouring, garlic purée, malted barley extract, onion powder.
Moving Mountains—Veggie Burger	Pea protein, wheat protein, soy protein, mushroom, onion, beetroot, coconut oil, oats, barley, soy protein isolate, gluten (wheat), flour (wheat), malt, methylcellulose, salt, vinegar, natural flavour, lemon juice, ascorbic acid, lactic acid, vitamin B12.
Quorn—Supreme Vegan Burger	Water, textured proteins (wheat gluten, pea protein, wheat starch, wheat flour, pea protein isolate), vegetable oils (sunflower, palm, coconut), mycoprotein (10%), natural flavours, beetroot juice (4%) (beetroot, lemon juice concentrate), stabiliser: methyl cellulose, potato protein, barley malt extract.
SoPeace—Burger	Rehydrated pea protein (45%), water, sunflower oil, seasoning (hydrolysed rapeseed protein, dextrose, vegetables (garlic, onion)), sugar, natural flavouring, herbs and spices (pepper, coriander, nutmeg), thickener (methyl cellulose), tapioca starch, onion, herbs, pea protein (2.2%), caramel, natural flavour, dextrose, vitamins (B2, B12), minerals (iron, zinc).
Vegafit—Gehaktschijf	38.4% rehydrated wheat protein, onion, sunflower oil, mustard (water, mustard seed, vinegar, salt, sugar, spices, herbs), potato protein, modified starch, salt, wheat flour, maltodextrin, sugar, soy hydrolysate, dextrose, yeast extract, garlic granulate, spices, potato fibre, wheat protein, thickener (carrageenan), wheat fibre, spices (celery), flavouring, barley malt, malt extract, caramelized sugar, iron, zinc, vitamin B12.
Vegetarische Slager—Mc2 No-Beef Burger	72% soy structure (water, soy protein, wheat protein, wheat starch), vegetable oils (sunflower, rapeseed), onion, free-range chicken EGG protein, onion extract, starch, herbs and spices, dextrose, salt, thickener: carrageenan, burnt sugar, flavour, yeast extract, palm fat powder, iron, vitamin B12.
VegiDeli—Quarter Pounder	Water, soy protein, onions, rapeseed oil, salt, thickener: carrageenan, methyl cellulose, natural flavourings, potato starch, dry yeast, sugar, spices, malt extract (contains barley), preservatives: sodium sorbate, onion powder, garlic powder.
Vivera—Vegetable Burger Patty	Rehydrated soy and wheat protein (72%), red onion, sunflower oil, natural flavourings, thickener (methylcellulose), hydrolysed wheat protein, potato starch, flavouring, dried glucose syrup, spices, sea salt, onion, potato fibres, dextrose, maltodextrin, garlic, onion extract, vitamins and minerals (iron, vitamin B12).

Table S2. Nutritional values per 100 g (from nutrition label) and pH (average from triplicate).

Chicken Pieces	Protein type	Raw/Cooked appearance	pH	Energy (kJ (kcal))	Protein (g)	Protein en%	Carbohydrates (g)	Of which sugars (g)	Fat (g)	Of which saturated (g)	Fibre (g)	Salt (g)	Vit. B12 (µg)	Iron (mg)
Real Chicken Pieces	-	R	6.2	480 (115)	24	83%	0	0	2.0	0.5	-	0.3	-	-
AH—Pieces Like Chicken	Wheat (25%), pea (12%)	C	6.1	760 (180)	23.0	51%	6.5	0.1	7.0	0.8	0.2	1.3	1.05	4.2
Food for Pro-gress—Oumph the Chunk	Soy (23%)	C	6.4	345 (82)	17.0	83%	0	0	0.4	0.0	5.1	0.6	-	3.3
Fry's—Chicken Style Strips	Wheat, soy	C	6.2	910 (218)	18.3	34%	7	0.2	11.8	1.4	5.4	1.3	-	-
Gold & Green—Pulled Oats	Oat, pea (21%), fava bean (12%)	C	6.4	897 (213)	30.0	56%	8.8	1	5.9	0.9	2.4	1.0	-	5.6
Greenway—Chick Pieces	Soy	C	6.0	521 (124)	17.8	57%	2.2	0.5	2.6	0.3	7.3	1.5	-	-
Naturli—Chick Free	Pea	C	5.9	546 (131)	21	64%	5.8	0.7	1.7	0.4	2.8	1.1	-	-
Quorn—Chicken Pieces	Mycopro-tein (94%), egg	C	5.5	475 (113)	15.3	54%	3.9	0.4	2.8	1.0	5.3	0.4	-	-
Valess—Fillet Pieces	Milk (76%), wheat	C	5.7	567 (135)	16.8	50%	3.8	1	4.8	0.8	4.9	1.7	-	2.2
Vantastic Foods—Chicken-Style Pieces	Soy (35%), wheat (20%)	R	6.5	758 (181)	15.0	33%	8.1	3.5	9.9	1.8	-	1.9	-	-
Vegetarische Slager—Kip-stuckjes	Soy (93%)	C	6.6	647 (154)	19.9	52%	5	0.5	4.4	0.5	7.6	1.9	-	-

Veggie Chef—Kipstukjes	Soy (93%)	C	6.8	504 (119)	19.4	65%	6.5	0.6	0.5	0.1	5.6	1.5	-	-
VegiDeli VBites—Meat-Free Chicken Pieces	Wheat, soy	C	5.8	804 (184)	26.6	58%	4	0.4	6.7	2.0	4.3	1.0	-	-
Vivera—Plant Chicken Pieces	Soy (93%)	C	6.9	500 (118)	19.0	64%	6.6	0.6	0.5	0.1	5.6	1.7	-	-
**Meat Analogue Pieces Average**	**-**	**-**	**6.2**	**633 (150)**	**19.9**	**56%**	**5.3**	**0.7**	**4.5**	**0.8**	**4.7**	**1.3**	**-**	**-**

**Burgers**	**Protein type**	**Raw/cooked appearance**	**pH**	**Energy (kJ (kcal))**	**Protein (g)**	**Protein en%**	**Carbohydrates (g)**	**Of which sugars (g)**	**Fat (g)**	**Of which saturated (g)**	**Fibre (g)**	**Salt (g)**	**Vit. B12 (μg)**	**Iron (mg)**
Ultimate Beef Burger (real beef)	-	R	6.1	970 (235)	20.0	34%	0.1	0	17.0	7.5	0.0	0.9	-	-
Albert Heijn—Burger Deluxe	Soy (53%), wheat (13%)	C	5.9	655 (155)	20.0	52%	4	1.5	5.0	0.6	7.5	1.3	0.4	2.1
Beyond Meat—Beyond Burger	Pea, rice	R	7.2	1107 (266)	18.0	33%	5.3	0	19.0	4.4	2.6	1.1	-	-
Fry's—Traditional Burgers	Soy, wheat	C	6.2	621 (148)	14.0	38%	7.4	0.3	5.6	0.7	6.2	2.0	-	-
Gardein—Ultimate Beefless Burger	Soy, wheat, pea	C	5.8	625 (149)	18.8	50%	5.2	1	5.2	0.0	3.1	0.9	-	2.2
Garden Gourmet—Incredible Burger	Soy (19%), wheat	R	5.9	817 (197)	14.4	29%	2.9	1.3	13.3	4.0	3.8	0.8	-	-
Greenway—Burger	Pea (22%), potato	R	6.3	771 (185)	14.0	30%	6.5	1	10.0	5.5	-	1.4	-	-

Linda McCartney—Quarter Pounder	Soy (58%)	R	6.1	921 (220)	17.3	31%	9.8	1.7	11.9	0.9	2.4	1.0	-	-
Moving Mountains—Veggie Burger	Pea, wheat, soy	R	5.7	1037 (250)	15.3	24%	7.2	0.8	17.6	15.7	5.8	1.3	3.1	-
Quorn—Supreme Vegan Burger	Wheat, pea, mycoprotein	R	6.0	1070 (255)	21.0	33%	10	3	14.0	3.0	3.1	1.2	-	-
SoPeace—Burger	Pea (45%)	R	6.3	978 (234)	16.5	28%	13.5	5.4	11.7	1.4	4.4	1.9	0.4	2.1
Vegafit—Gehaktschijf	Wheat (38%), potato	C	5.6	1090 (260)	15.5	24%	15.6	3.5	15.6	1.6	4.5	2.0	0.38	2.1
Vegetarische Slager—Mc2 No-Beef Burger	Soy, wheat	C	6.0	709 (170)	17.0	40%	6.5	1.5	8.6	1.4	<0.5	1.2	0.38	2.1
VegiDeli—Quarter Pounder	Soy	C	6.1	791 (183)	19.3	42%	6.2	1.2	8.7	0.7	-	1.3	-	-
Vivera—Vegetable Burger Patty	Soy, wheat	C	5.8	686 (164)	17.0	41%	8.9	1.8	5.4	0.6	6.0	1.2	0.38	2.1
**Meat Analogue Burgers Average**	**-**	**-**	**6.1**	**848 (203)**	**17.0**	**35%**	**7.8**	**1.7**	**10.8**	**2.9**	**4.5**	**1.3**	**-**	**-**

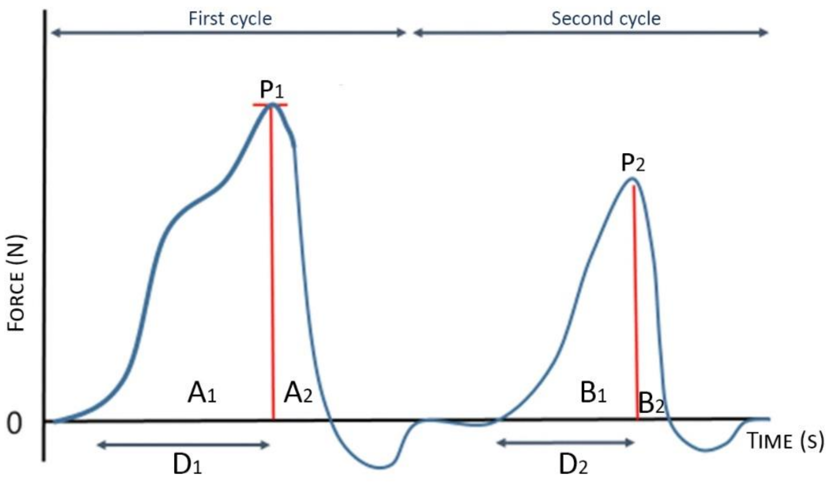

Figure S1. Stress–strain curve for TPA test. Letters indicate relevant parameters for texture attribute calculations.

Product	Raw and cooked	Product	Raw and cooked
**Chicken pieces samples**		**Burger samples**	
Real Chicken Pieces (Albert Heijn)	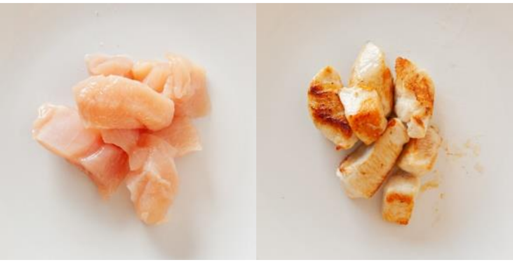	Ultimate Beef Burger (real beef)	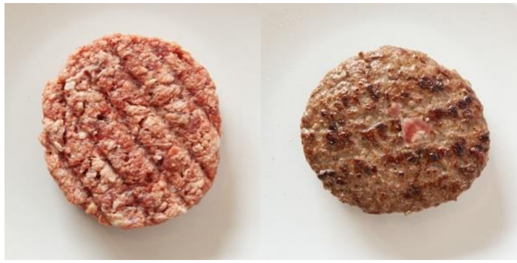
Albert Heijn—Pieces Like Chicken	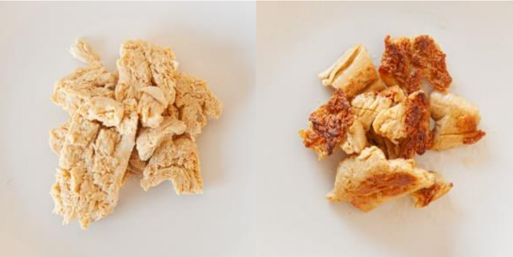	Albert Heijn—Burger Deluxe	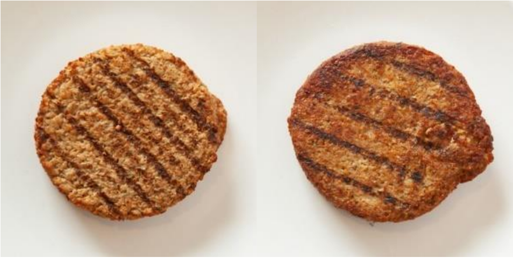
Food for Progress—Oumph the Chunk	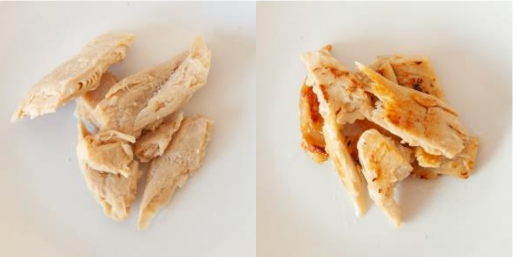	Beyond Meat—Beyond Burger	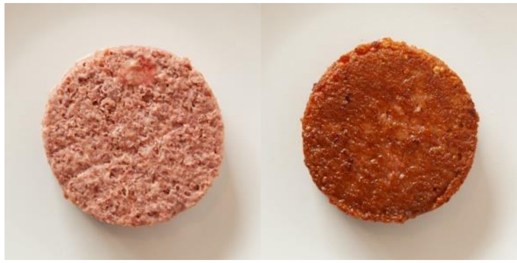
Fry's—Chicken Style Strips	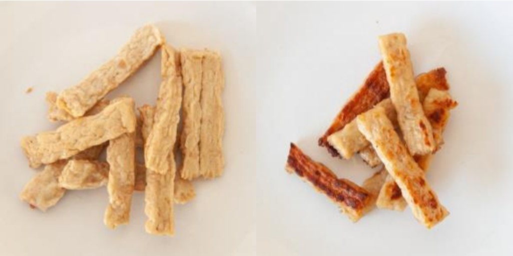	Fry's—Traditional Burgers	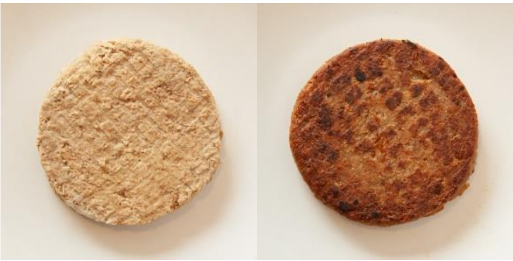

Gold & Green—Pulled Oats	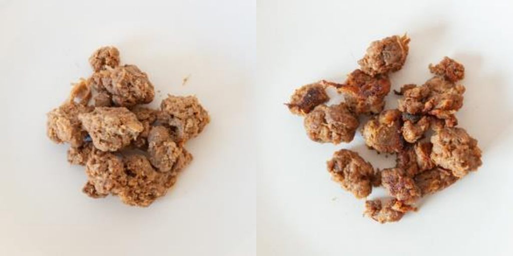	Gardein—Ultimate Beef-less Burger	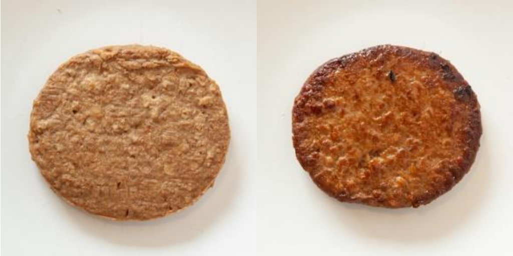
Greenway—Chick Pieces	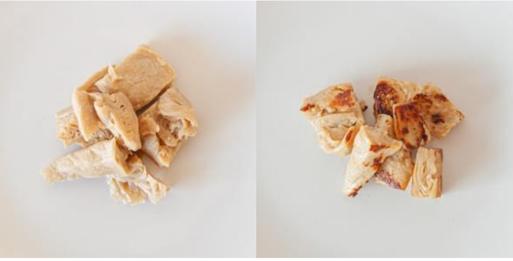	Garden Gourmet—In-credible Burger	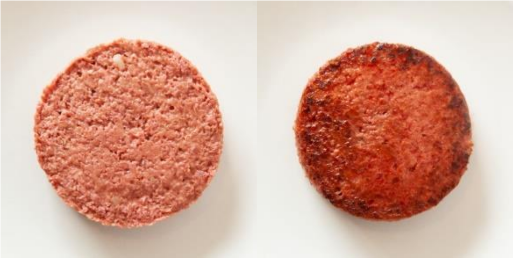
Naturli—Chick Free	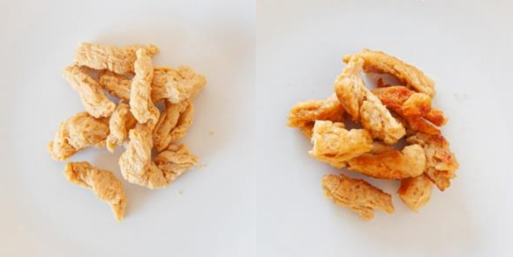	Greenway—Burger	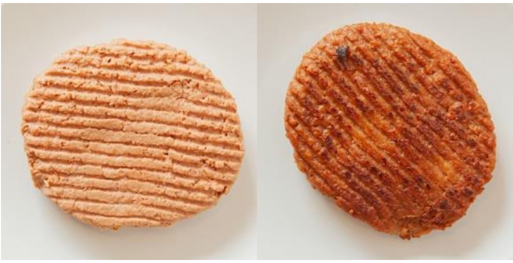
Quorn—Chicken Pieces	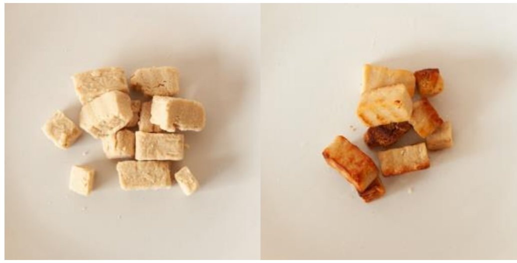	Linda McCartney—Quarter Pounder	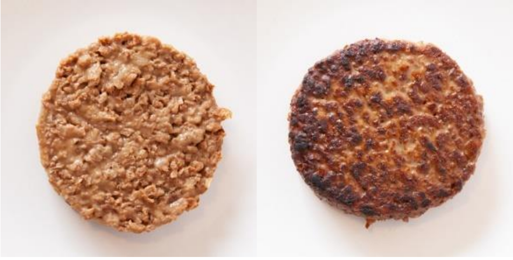

Valess—Fillet Pieces

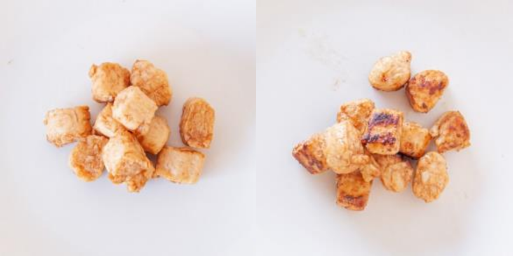

Vantastic Foods—Chicken-Style Pieces

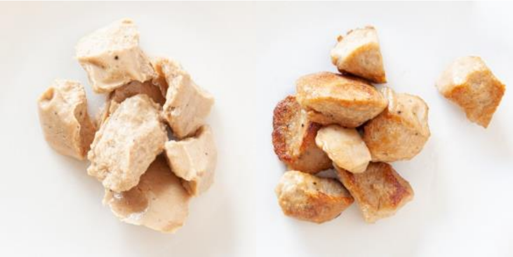

Vegetarische Slager—Kip-stuckjes

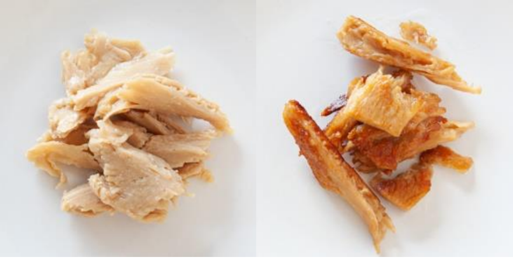

Veggie Chef—Kipstukjes

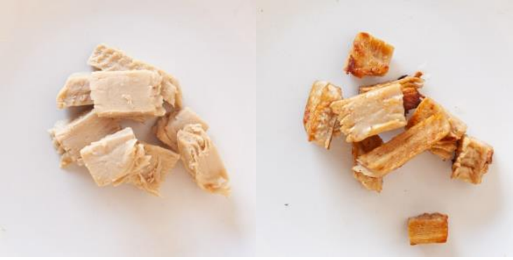

Moving Mountains—Veggie Burger

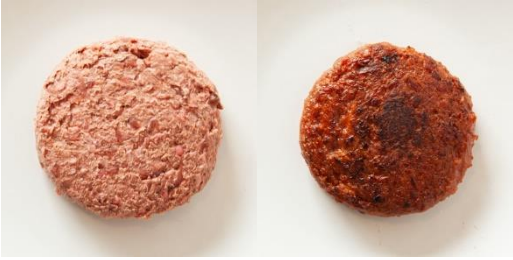

Quorn—Supreme Vegan Burger

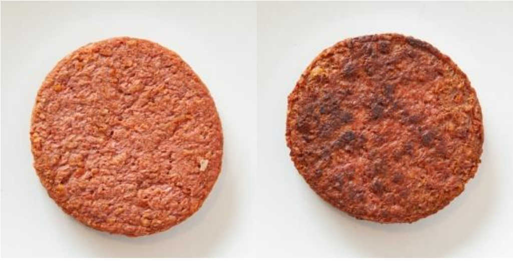

SoPeace—Burger

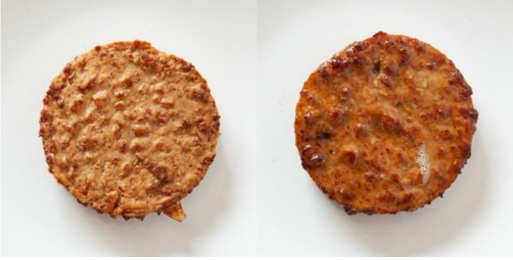

Vegafit—Gehaktschijf

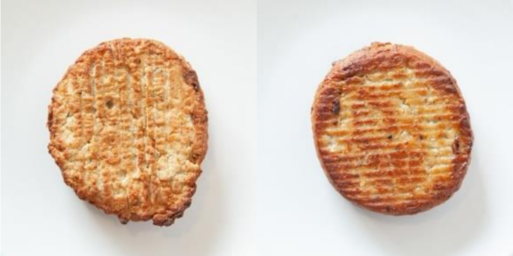

VegiDeli VBites—Meat-Free Chicken Pieces

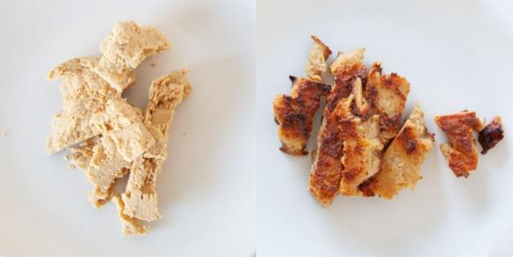

Vivera—Plant Chicken Pieces

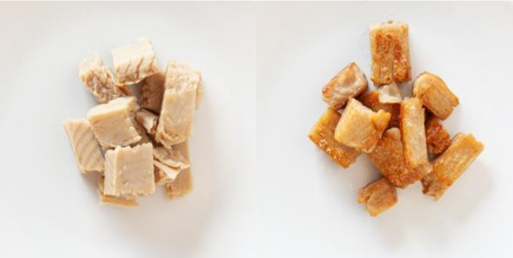

Vegetarische Slager—Mc2 No-Beef Burger

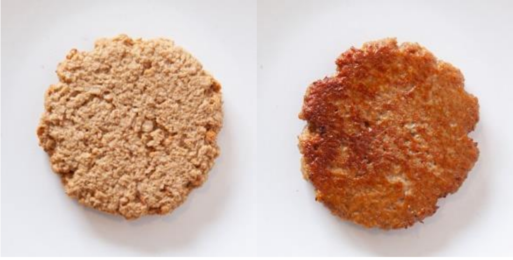

VegiDeli—Quarter Pounder

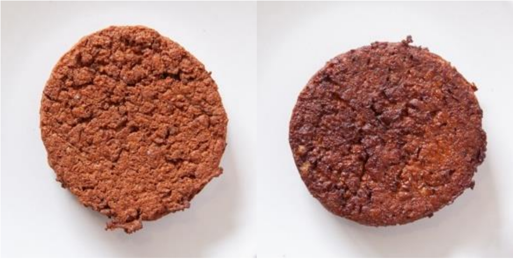

Vivera—Vegetable Burger Patty

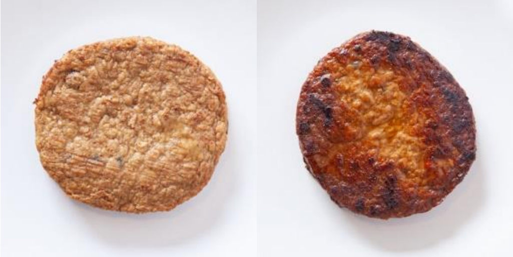

Figure S2. Photos of raw and cooked samples, top view.

Flavour liking vs. flavour attributes

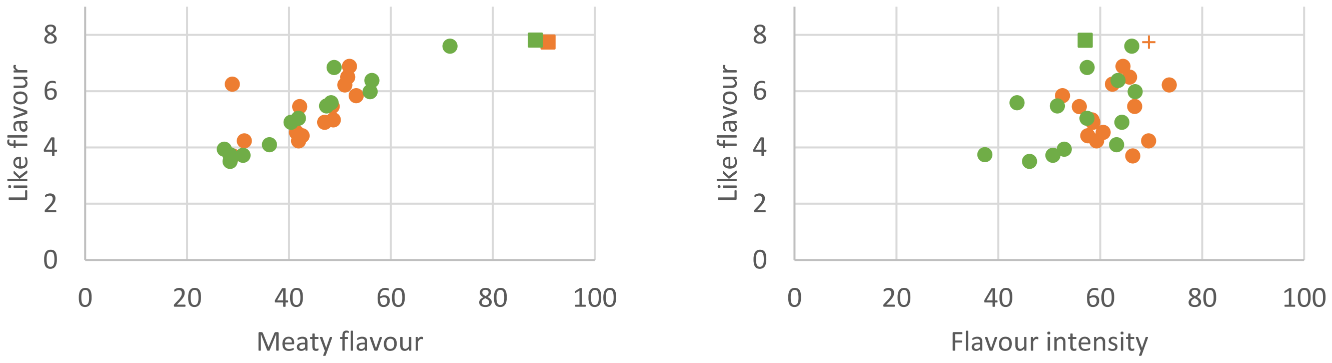

Texture liking vs. texture attributes

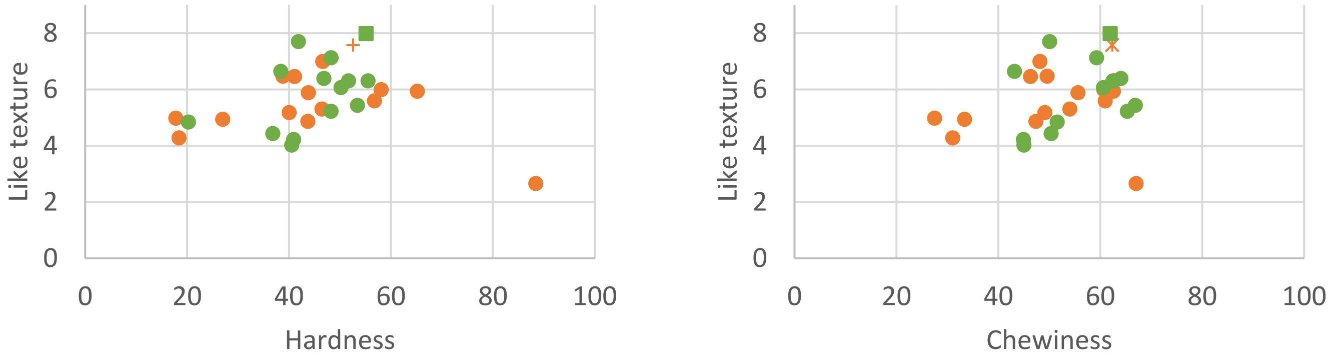

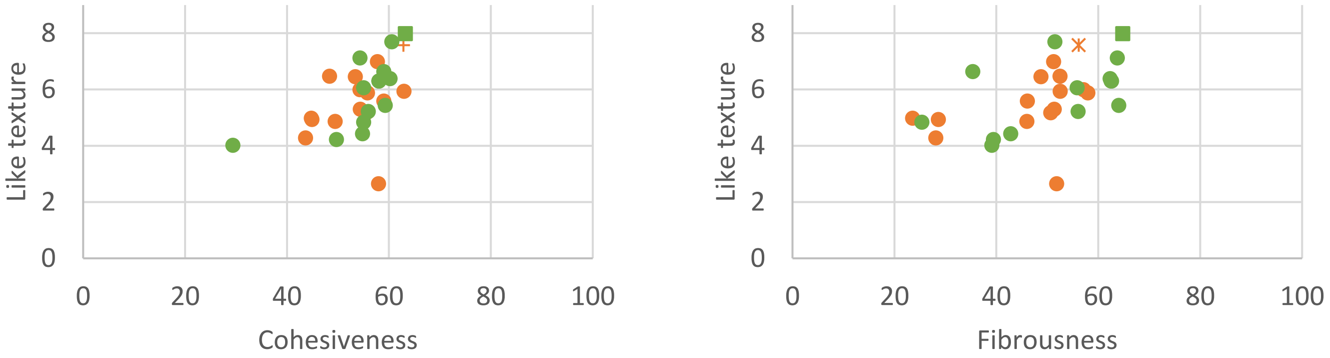

Texture liking vs. mouthfeel attributes

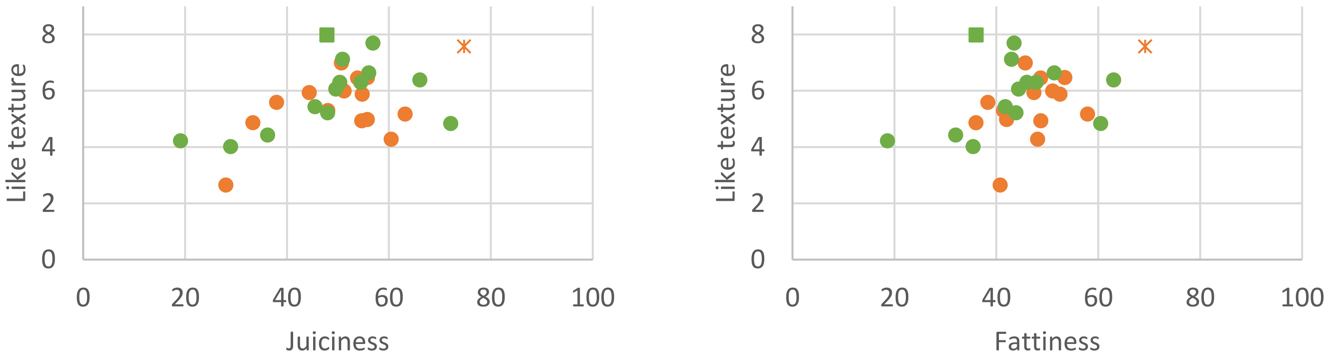

Texture like meat vs. juiciness

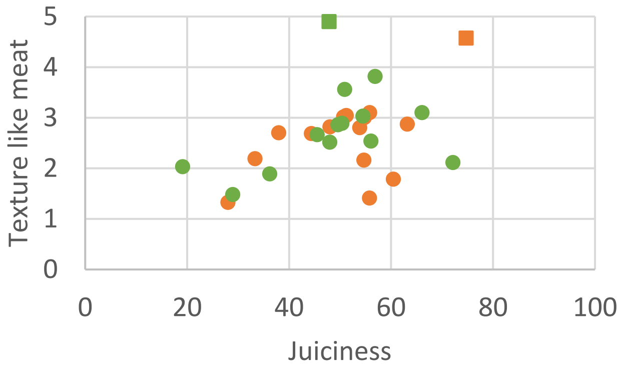

Figure S3. Correlation scatterplots of flavour and texture liking (scale: 1–9) and attributes (scale: 0–100), and 'texture like meat' (scale: 1–5) versus juiciness (scale: 0–100). Green datapoints are chicken pieces; orange datapoints are burgers. Dots are meat analogues; squares are meat samples.
